# Retraction Note: Granulocyte colony stimulating factor versus human chorionic gonadotropin for recurrent implantation failure in intra cytoplasmic sperm injection: a randomized clinical trial

**DOI:** 10.1186/s12884-025-08366-6

**Published:** 2025-10-15

**Authors:** Mohamed Sobhy Bakry, Elsayed Eldesouky, Moatazza Mahdy Alghazaly, Elsayed farag, Eslam Elsayed Kamal Sultan, Hossam Elazzazy, Attia Mohamed, Soliman Mohamed Said Ali, Assem Anwar, Asmaa Ahmed Elrashedy, Mohamed Abdelmonem, Mohamed Abd-ElGawad, Almandouh H. Bosilah

**Affiliations:** 1 Department of Obstetrics and Gynecology, Faculty of Medicine, Fayoum University, Fayoum, Egypt; 2 Department of Obstetrics and Gynecology, Faculty of Medicine, Alazhar University, Cairo, Egypt; 3 Department of Obstetrics and Gynecology, Faculty of Medicine Girls, Alazhar University, Cairo, Egypt; 4 Department of Obstetrics and Gynecology, Faculty of Medicine, Alazhar University, Domiata, Egypt; 5 Faculty of Medicine, Kafr El-Shaikh University, Kafr El-Shaikh, Egypt; 6 Faculty of Medicine, Fayoum University, Fayoum, Egypt; 7 Department of Obstetrics and Gynecology, Faculty of Medicine, Domiata University, Domiata, Egypt

**Retraction Note: BMC Pregnancy and Childbirth 22, 881 (2022)**

**https://doi.org/10.1186/s12884-022-05098-9**

The Editor has retracted this article. After publication, concerns were raised regarding the data in the paper. Upon reviewing the raw data provided by the authors, there appear to be duplicate entries along with inconsistencies such as cases with more MII oocytes than total oocytes retrieved. Authors Mohamed Abd-ElGawad, Mohamed Abdelmonem Kamel and Asmaa Ahmed Elrashedy confirmed there was issue with the data and asked the Editor to retract the paper. Asmaa Ahmed Elrashedy and Mohamed Abd-ElGawad agree tp this retraction. Mohamed Sobhy Bakry, Elsayed Eldesouky, Moatazza Mahdy Alghazaly, Elsayed farag, Eslam Elsayed Kamal Sultan, Hossam Elazzazy, Attia Mohamed, Soliman Mohamed Said Ali, Assem Anwar, Mohamed Abdelmonem and Almandouh H. Bosilah did not reply whether they agree to this retraction.

